# NeuN/Rbfox3 Nuclear and Cytoplasmic Isoforms Differentially Regulate Alternative Splicing and Nonsense-Mediated Decay of Rbfox2

**DOI:** 10.1371/journal.pone.0021585

**Published:** 2011-06-29

**Authors:** B. Kate Dredge, Kirk B. Jensen

**Affiliations:** School of Molecular and Biomedical Science, The University of Adelaide, Adelaide, Australia; Centre de Regulació Genòmica, Spain

## Abstract

Anti-NeuN (Neuronal Nuclei) is a monoclonal antibody used extensively to specifically detect post-mitotic neurons. Anti-NeuN reactivity is predominantly nuclear; by western it detects multiple bands ranging in molecular weight from 45 kDa to >75 kDa. Expression screening putatively identified R3hdm2 as NeuN; however immunoprecipitation and mass spectrometry of the two major NeuN species at 45–50 kDa identified both as the RNA binding protein Rbfox3 (a member of the Fox family of alternative splicing factors), confirming and extending the identification of the 45 kDa band as Rbfox3 by Kim *et al*. Mapping of the anti-NeuN reactive epitopes in both R3hdm2 and Rbfox3 reveals a common proline- and glutamine-rich domain that lies at the N-terminus of the Rbfox3 protein. Our data suggests that alternative splicing of the Rbfox3 pre-mRNA itself leads to the production of four protein isoforms that migrate in the 45–50 kDa range, and that one of these splicing choices regulates Rbfox3/NeuN sub-cellular steady-state distribution, through the addition or removal of a short C-terminal extension containing the second half of a bipartite hydrophobic proline-tyrosine nuclear localization signal. Rbfox3 regulates alternative splicing of the Rbfox2 pre-mRNA, producing a message encoding a dominant negative form of the Rbfox2 protein. We show here that nuclear Rbfox3 isoforms can also enhance the inclusion of cryptic exons in the Rbfox2 mRNA, resulting in nonsense-mediated decay of the message, thereby contributing to the negative regulation of Rbfox2 by Rbfox3 through a novel mechanism.

## Introduction

NeuN (Neuronal Nuclei) is a protein ‘marker’ detected exclusively in post-mitotic neurons that was initially identified through an immunologic screen to produce neuron-specific antibodies. The result of this screen, in which mice immunized with mouse brain nuclei extracts were used to derive a panel of monoclonal antibodies (mAbs), was mAb A60 (anti-NeuN), which recognizes at least two major protein species migrating at approximately 45–50 kDa, by western of brain extracts [1], [2]. In addition to the 45–50 kDa NeuN doublet, some cell lysate preparations show additional reactive bands at ∼66 kDa, and between ∼70–90 kDa by western. Immunohistochemically, NeuN is detected only in mature neurons and is absent from neural progenitors, glia, oligodendrocytes and astrocytes [2]. In general, NeuN reactivity is predominantly nuclear, although it can also be detected in the cytoplasm of many neuronal cell types [2], [3]. It has been hypothesized that NeuN may be required for the maintenance of the post-mitotic state, or during the process of axonogenesis [4], but these questions have been impossible to address without the molecular characterization of NeuN.

Despite the widespread use of the anti-NeuN mAb, the identity of the NeuN protein remained elusive for 17 years. As a result, almost nothing was known about the function of NeuN protein beyond a demonstrated ability to bind DNA *in vitro* [2]. Kim *et al.* [5] and work presented here identify NeuN as Rbfox3. The Fox (feminizing on X) proteins are a highly conserved family of tissue-specific splicing regulators that each harbor a single RNA-recognition motif (RRM)-type RNA binding domain. Rbfox1 (A2bp1) is expressed in neurons, skeletal muscle and heart [6]–[9]. Rbfox2 (Rbm9) is expressed in ovary, whole embryo, and human embryonic cell lines in addition to neurons and muscle [6], [10]. Rbfox3 (Hrnbp3, D11Bwg0517e) message is detected exclusively in post-mitotic regions of embryonic mouse brain [11]. Fox proteins have been shown to regulate a large number of brain and muscle-specific splice choices via binding to the hexanucleotide UGCAUG, including: exon EIIIB of fibronectin; exon N1 of c-*src*; and calcitonin/CGRP [6], [8], [12].

All Fox family members are subject to alternative splicing; we show here that the major NeuN products seen by Western are created by two separate alternative splicing events that create Rbfox3 protein variants with either nuclear or cytoplasmic steady-state distribution. We have tested three individual Rbfox3 proteins in alternative splicing assays and find that all of these Rbfox3 protein isoforms repress inclusion of the alternative RRM exon, exon 6, of Rbfox2, giving rise to a variant of Rbfox2 without a functional RRM. An equivalent alternatively spliced exon is also found in mammalian Rbfox1 and Rbfox3, as well as in the *Drosophila* Fox homolog, and it has recently been demonstrated that these FoxΔRRM isoforms encode proteins that can act as dominant negative splicing factors [13]. Regulation of this splice choice represents one mechanism for modulation of Fox protein function in cells expressing multiple Fox family members [13].

A number of splicing factors, including SC35 and polypyrimidine tract binding protein (PTB/Ptbp1), have been shown to autoregulate their expression by regulating alternative splicing of their own pre-mRNA to enhance the production of mRNA isoforms that are subject to nonsense-mediated decay (NMD) [14], [15]. NMD is a surveillance pathway that is triggered in mammalian cells when an mRNA contains a nonsense codon more that 50–55 nucleotides upstream of an exon-exon junction [16]. Our alternative splicing assays also reveal evidence of a second, novel mechanism of Fox family cross-regulation through alternative splicing associated nonsense-mediated decay (NMD).

## Results

**Figure 1:**
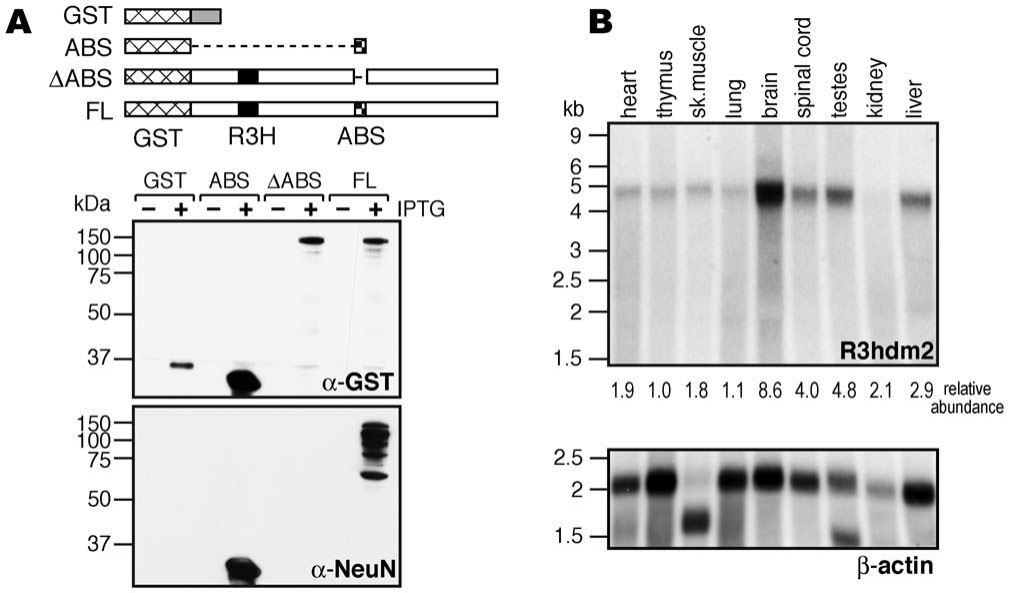
R3hdm2 reacts with anti-NeuN antibody. A. The depicted GST-fusion proteins were expressed in bacteria and cell lysates analyzed by western. The upper western panel (anti-GST) confirms that proteins of the expected sizes were expressed after IPTG induction. ABS = antibody binding site; FL = full-length R3hdm2; the R3H domain is also shown. The membrane was then stripped and reprobed with anti-NeuN antibody (lower panel), which indicates that the defined ABS region is both necessary and sufficient for anti-NeuN recognition of R3Hdm2. B. Northern of poly(A)^+^ RNA isolated from adult mouse tissues and probed for R3hdm2. The level of R3hdm2 expression was normalised to β-actin (lower panel) for each tissue and calculated relative to the expression level in thymus.

We initially sought to identify NeuN using a lambda phage library-screening approach. We used anti-NeuN to screen a cDNA expression library derived from P0 mouse spinal cord poly(A)^+^ RNA and isolated four independent, overlapping clones encoding the poorly characterized protein R3hdm2 (KIAA1002). This protein contains only one known protein motif, an R3H domain, which has been shown to bind single-stranded nucleic acid [17]. Deletion mapping of the smallest positive clone was used to narrow the anti-NeuN antibody-binding site (ABS) to a 46 amino acid domain necessary and sufficient for recognition of GST-R3hdm2 fusions by western with anti-NeuN (Figure 1A).

While our data demonstrate that anti-NeuN is able to recognize R3hdm2 *in vitro*, the full-length clones isolated from our screen encode proteins of ∼110 kDa, clearly much larger than the classic 45–50 kDa NeuN doublet seen by western. Furthermore, northern analysis (Figure 1B) shows that R3hdm2 message, while enriched in mouse brain, spinal cord and testes, is also present in lower levels in other tissues. Thus, while the R3hdm2 mRNA is most abundant in brain, tissue regulation of the message cannot by itself account for the exquisite neuronal specificity of anti-NeuN protein recognition. Given the discrepancies in the molecular weight and expression properties of R3hdm2 and NeuN, we employed an alternative approach in an effort to unambiguously identify NeuN.

### Identification of the NeuN doublet as Rbfox3 by immunoprecipitation and mass spectrometry

**Figure 2:**
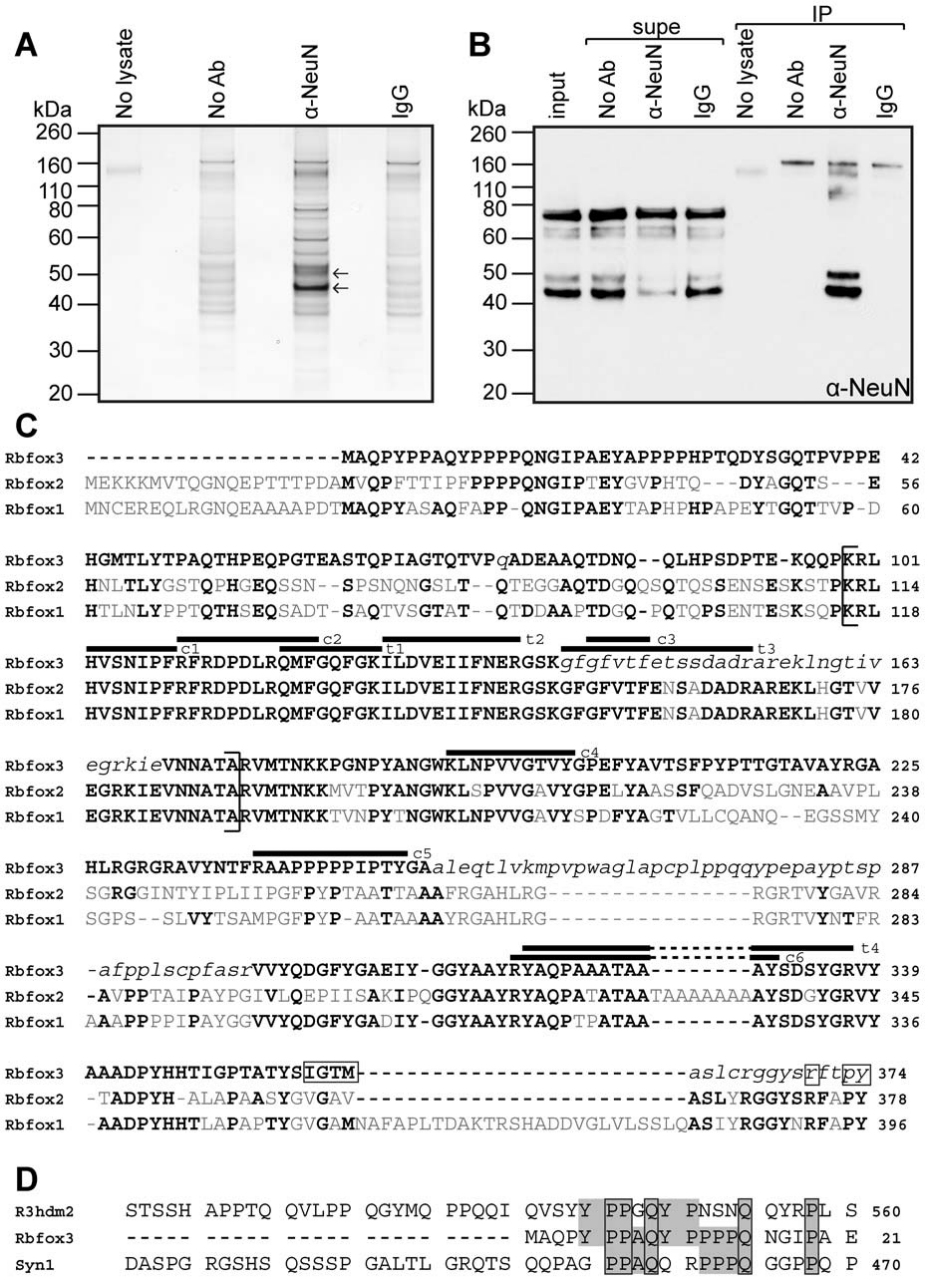
Immunoprecipitation and mass spectrometry identify Rbfox3 as the target of anti-NeuN. A. Silver stain of proteins immunoprecipitated from P20 mouse brain crude nuclear extract using anti-NeuN, and separated by non-reducing SDS-PAGE. No antibody, ands mouse IgG were used as IP controls; the no lysate sample was used to check the integrity of anti-NeuN cross-linking (covalent coupling) to the beads. Arrows point to the bands analyzed by mass spectrometry. B. Western confirms the specificity of the anti-NeuN immunoprecipitation. The “classic” NeuN doublet species below 50 kDa were efficiently immunoprecipitated, and depleted from the supernatant (supe), while the upper immunoreactive bands were not. C. Alignment of the mouse Fox protein family. Accession numbers of the sequences shown are; Rbfox1 (Fox-1, A2bp1, Hrnbp1) NP_067452, Rbfox2 (Fox-2, Rbm9, Fxh) NP_001104299, Rbfox3 (Fox-3, Hrnbp3) NP_001034256. Amino acids that differ in sequence from Rbfox3 are shown in grey. Lower case italics denote regions of Rbfox3 that may be absent due to alternative splicing. The RRM domain (as defined by UniProt) is contained within parentheses. Black lines above the sequence mark peptides identified by MS after in-gel digestion with chymotrypsin (c) or trypsin (t). Boxes surround Rbfox3 residues that match the hPY-NLS consensus. D. Alignment of the anti-NeuN ABS from R3hdm2 with the N-terminus of Rbfox3 and the predicted antibody-binding region of Syn1 from Kim *et al.* [5]. Amino acids identical to those in Rbfox3 are shaded grey, boxes outline amino acids that are identical across all three proteins.

To directly identify the major 45–50 kDa NeuN doublet, we immunoprecipited (IPed) NeuN with anti-NeuN mAb, separated the eluate by SDS-PAGE and subjected excised protein bands to mass spectrometry (MS). To avoid interference of IgG heavy-chain, which runs at a similar molecular weight to NeuN on reducing SDS-PAGE, we crosslinked the anti-NeuN antibody to protein-A sepharose beads, and ran non-reducing gels. We saw antibody-dependent enrichment of bands corresponding in size to the NeuN doublet by silver-stain and western of IPed fractions (Figure 2A & 2B). While a number of other proteins were also specifically IPed under these conditions, since the majority of these bands do not appear to be recognized by anti-NeuN western, they may represent NeuN-interacting proteins, and were not analyzed further. However, there were a series of bands ∼100–110 kDa in size that were revealed by western as specifically IPed by anti-NeuN (Figure 2B) and these are consistent in size with isoforms of R3hdm2. Conversely, several species between 60 and 80 kDa that are clearly detected by western were neither depleted from the extract nor present in the IPed fraction. The experiment shown in Figures 2A and 2B was performed using a crude nuclear preparation that was not further purified to remove cellular membranes; based on the work of Kim *et al.* [5], we suspect that the largest of these (if not several of them) is likely to be synapsin 1, a synaptic vesicle-associated protein which they showed can be detected using anti-NeuN by western.

The bands corresponding to the NeuN doublet at 45–50 kDa were cut from the silver-stained gel and subsequently digested with chymotrypsin, followed by tandem MS analysis of the resulting peptides. The choice to use chymotrypsin rather than the more routinely used trypsin was made because our lambda-screen candidate R3hdm2 contains a striking paucity of predicted tryptic cleavage sites, particularly surrounding the mapped antibody binding site (ABS). We reasoned that if NeuN was indeed R3hdm2, or a portion thereof, a tryptic digest could fail to produce peptides suitable for an identification to be made using MS. While the MS analysis was successful with chymotrypsin, no peptides corresponding to R3hdm2 were detected. Instead, six peptides were detected that correspond to Rbfox3 (black lines, c1–c6, in Figure 2C), a recently characterized member of the Fox family of alternative splicing regulators. Of these, three peptides (c1–c3) were derived from portions of the protein that are identical in the 3 members of the Fox protein family. The remaining three peptides (c4–c6) correspond only to Rbfox3 and thus allowed for unequivocal identification of the protein. Peptides c1 and c2 were seen in the analysis of the lower band only, whereas peptides c3–c6 were detected after digest of both the upper and lower bands of the NeuN doublet. Thus we provide direct evidence that the upper, 50 kDa band of the NeuN doublet is Rbfox3 in addition to independently confirming the mass–spectrometric identification of the 45 kDa band performed by Kim *et al.* [5].

**Figure 3:**
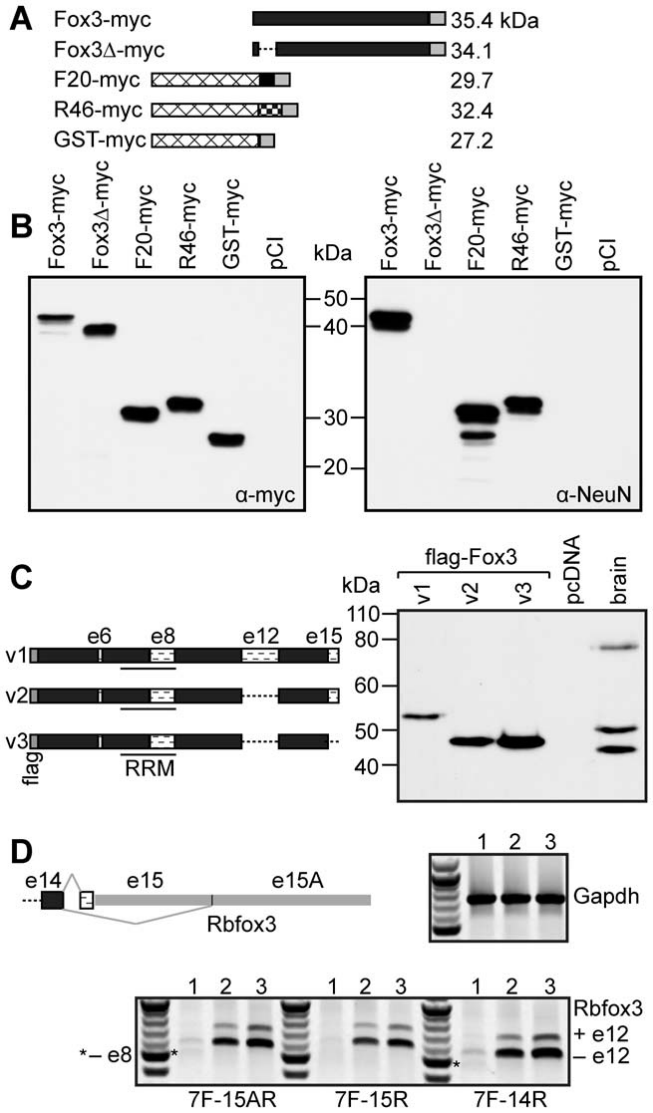
The anti-NeuN epitope of Rbfox3 maps to the extreme N-terminus of the protein. A,B. The proteins depicted in A were expressed in 293T cells and analyzed by western with anti-myc-tag (left panel), or anti-NeuN antibodies on duplicate blots. Anti-NeuN recognises both F20 and R46, and amino acids 5–20 (deleted in Fox3Δ-myc) are necessary for recognition of full-length Rbfox3 by anti-NeuN. C. Three Rbfox3 splice variants were expressed in HeLa cells and compared to NeuN from mouse brain by western with anti-NeuN. Variant 1 (v1) corresponds to NP_001034256, v2 to NP_001034257, and v3 to NP_001020102. Exon numbers are as described in [13]. D. RT-PCR of total RNA from P19 cells prior to neuronal induction (lanes 1), P19 cells 7 days after neuronal induction with RA (lanes 2), and P10 mouse brain (lanes 3). For Rbfox3, 3 reverse primers were used to assess mRNAs that encode the short C-terminus (or -IGTM protein isoforms, 15A-R), the longer, -FTPY isoforms (15-R), or all mRNAs (14-R). A common forward primer to exon 7 (7-F) was used for all. Gapdh was used as a loading control.

A further experiment using purified mouse brain nuclear extract and in-gel tryptic digest of the lower NeuN band revealed four peptides derived from Rbfox3. Again, two of these (t3 and t4) are unique to Rbfox3, while the remaining two are common to the Fox family. The Rbfox3 pre-mRNA is alternatively spliced in a number of regions resulting in changes to the encoded protein (see Figure 3C and [13]). From our MS data, we were able to determine that *both* of the NeuN doublet bands are indeed Rbfox3, and both include a cassette exon, exon 8, which encodes the C-terminal half of the RRM domain. Thus, while we were able to unambiguously identify both of the immunoprecipitated bands as Rbfox3 with full-length RRMs, we found no other peptides that corresponded to alternatively spliced regions. As a consequence, the MS data did not provide an explanation for the ∼5 kDa difference in molecular weight between the upper and lower NeuN bands.

### The anti-NeuN epitope resides at the extreme N-terminus of Rbfox3

We next aligned our mapped anti-NeuN epitope from R3hdm2 with the Rbfox3 protein sequence in order to predict the likely anti-NeuN epitope in Rbfox3. Figure 2D shows that a portion of the 46 amino acid R3hdm2 ABS is identical in 8 of 15 amino acids to the N-terminal region of Rbfox3. Of these, 5 residues are also identical to the region of synapsin 1a predicted to be a weak epitope for anti-NeuN by Kim *et al.* [5]. To directly test whether anti-NeuN recognizes Rbfox3 via this region, we created the deletion construct Fox3Δ-myc, which lacks Rbfox3 amino acids 5–20 (replaced with the sequence GSAP), and the construct F20-myc, which harbors Rbfox3 amino acids 2–20 immediately C-terminal of glutathione S-transferase (GST) (Figure 3A). We transfected these constructs into 293T cells and compared their detection with anti-NeuN by western to a full-length Rbfox3 (Fox3-myc), the construct R46-myc (a GST-R3hdm2 ABS fusion) and GST alone. All of the tested constructs contain a myc-tag to allow detection of the proteins by western using an anti-myc antibody. Figure 3B shows that the N-terminal 21 amino acids of Rbfox3 are necessary and sufficient for recognition of the protein by anti-NeuN. Taking into account the relative amounts of protein seen with anti-myc staining, anti-NeuN appears to recognize full-length Fox3-myc slightly better than F20-myc, and F20-myc slightly better than R46-myc.

### Alternative splicing of Rbfox3 pre-mRNA can account for the pattern of NeuN bands seen by western

The Rbfox3 mRNA exists as multiple splice variants (see Figure 3C and [13]). We cloned the coding sequence of the three most common mouse Rbfox3 mRNA variants (variants 1–3 in the NCBI database) downstream of a flag-tag sequence and expressed them in HeLa cells. Variant 1 includes a 47 amino acid protein-coding domain derived from an extended exon 12, and a 14 amino-acid C-terminal protein coding domain derived from exon 15, both of which are created by the use of alternative 3′ splice sites in the respective upstream introns. Variant 2 lacks the additional exon 12 protein-coding sequence found in variant 1, but contains the same C-terminal protein-coding sequence as variant 1. Variant 3 lacks both of these additional protein-coding domains. The calculated molecular weights of these Rbfox3 variants (without tags) are: variant 1, 40.6 kDa; variant 2, 35.5 kDa; variant 3, 34.1 kDa.

We had previously noticed that the Fox3-myc protein migrated on SDS-PAGE gels at a molecular weight slightly greater than its calculated molecular weight. When we analyzed the Rbfox3 protein variants by SDS-PAGE and detection with anti-NeuN, flag-Fox3v1 ran at a slightly greater molecular weight than the upper NeuN band from mouse brain, while variants 2 and 3 ran at similar but not identical positions in the gel, both at a slightly greater molecular weight than the lower NeuN band (Figure 3C). If one accounts for both the general Rbfox3 protein migration discrepancy and the added molecular weight of the flag-tag, the gel migration behavior of the Rbfox3 protein variants strongly suggests that the 45 and 50 kDa NeuN bands represent ‘−exon 12’ and ‘+exon 12’ variants respectively. Furthermore, close inspection of anti-NeuN westerns reveals that the antibody actually recognizes a “doublet of doublets” ([1], [2] and our data not shown), where both the 45 and 50 kDa ‘bands’ are actually composed of two individual protein species differing very slightly in molecular weight. This data suggests that both the 45 and 50 kDa ‘bands’ are composed of a mixture of Rbfox3 proteins alternatively spliced at exon 15. Thus, alternative splicing of the Rbfox3 message in exon 12 and exon 15 produces four protein isoforms that can account for the 45–50 kDa doublet of doublets that are detected with anti-NeuN. Two other regions of the Rbfox3 coding sequence can also be alternatively spliced. Exon 6 contains two alternative 3′ splice sites just 3 nt apart, creating proteins +/− a single glutamine residue, which were not analyzed in this study. Exon 8, which encodes 31 amino acids (3.4 kDa) of the RRM domain, is a cassette exon that can be included or skipped; the MS data demonstrated that both the 45 and 50 kDa NeuN/Rbfox3 bands include exon 8, so alternative splicing of this exon cannot account for the migration difference between these two ‘bands.’

It has been well documented that the NeuN protein is expressed in P19 embryonic carcinoma cells after neural induction with retinoic acid, but cannot be detected in uninduced P19 cells [2], [5]. We examined Rbfox3 mRNA expression in these two P19 differentiation states by RT-PCR, along with mouse brain tissue as a control. To carry out this analysis, we used a forward primer to exon 7 (7F) and three reverse primers, 15AR, 15R and 14R (see the Rbfox3 pre-mRNA diagram in Figure 3D): the primer to exon 15A (15AR) amplified mRNA variant 3 and other isoforms lacking the 14 amino acid C-terminal peptide extension; the primer to exon 15 (15R) amplified message variants 1 and 2 and any other isoforms encoding the C-terminal extension; finally, the primer to exon 14 (14R) was used as a control to amplify all Rbfox3 mRNA isoforms, but gives no information about splicing of the final intron and thus the C-terminal composition of the protein products. As shown in Figure 3D, very little Rbfox3 message was detected in uninduced P19 cells (lanes 1), and the modest amount of product that was amplified corresponds to Rbfox3 protein with the shorter C-terminus. There was also a trace amount of product corresponding to messages lacking exon 8 in these cells. As stated above, skipping of exon 8 leads to a 31 amino acid deletion within the RRM domain. EST evidence supports the presence of this isoform in mouse retina, however, we have no evidence that Rbfox3ΔRRM protein, which should retain reactivity with anti-NeuN, is expressed in detectable levels in any tissues or cell lines tested so far.

Induction of P19 cells with retinoic-acid results in a large increase in the abundance of all Rbfox3 message variants as assayed by RT-PCR (lanes 2). The complement of transcripts seen in induced P19 cells is very similar to that seen in P10 mouse brain (lanes 3) in which the majority of transcripts lack exon 12, and both exon 15 variants are present. Taken together with our MS and western data, the RT-PCR studies support our finding that alternative splicing of exons 12 and 15 is the basis of the doublet of doublets observed by western analysis of mouse brain and induced P19 cells with anti-NeuN.

### Rbfox3 sub-cellular localization is regulated by alternative splicing

**Figure 4:**
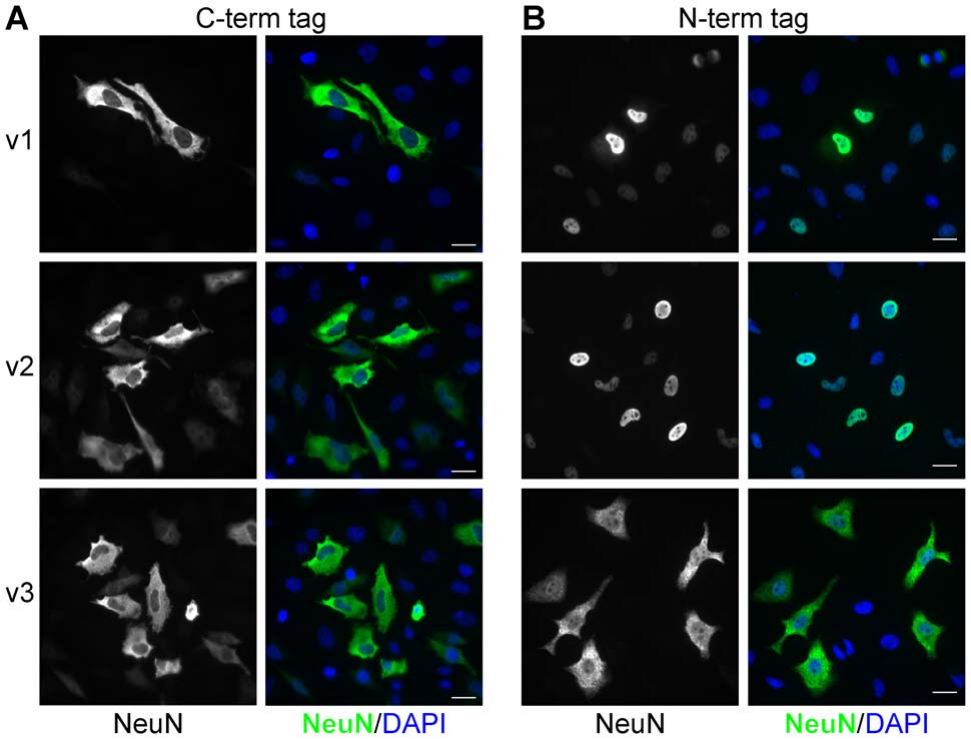
The C-terminus of Rbfox3 specifies sub-cellular location. Rbfox3 variants 1, 2 and 3 harboring C-terminal myc-tags (A), or N-terminal flag-tags (B) were expressed in HeLa cells and stained with anti-NeuN and DAPI. All C-terminally tagged proteins displayed predominantly cytoplasmic localization, whereas N-terminally tagged Fox3v1 and v2 displayed the expected nuclear localization. Scale bars indicate 50 µm.

NeuN is reported to be predominantly nuclear, although cytoplasmic staining has been observed in subsets of neurons [2], [3]. To investigate their sub-cellular localization, we transfected vectors encoding the Rbfox3 variants 1–3 into HeLa cells and analyzed them by immunofluorescence with anti-NeuN. We initially used constructs harboring C-terminal myc-tags and found that all three Rbfox3 protein variants localized to the cytoplasm, in contrast to the expected nuclear localization (Figure 4).

Interestingly, the Rbfox1 pre-mRNA can also be alternatively spliced to produce Rbfox1 protein variants with unique C-termini; Rbfox1 isoforms that end in the sequence –FAPY are predominantly nuclear at steady-state, whereas isoforms ending in –TALVP are predominantly cytoplasmic [18]. Recently, it was noted that the –FAPY C-termini variants of Rbfox1 and −2 match the two-part consensus hydrophobic proline-tyrosine nuclear localization sequence (hPY-NLS); namely φG/A/SφφX_(11–13)_PY and R/H/KX_(2–5)_PY where φ represents a hydrophobic side chain [19], [20]. The C-terminal extension variants of Rbfox3 also satisfy this two-part NLS signal (highlighted in Figure 2C); Rbfox3 variants without the C-terminal extension harbor only the first half of the bipartite NLS signal. Proteins harboring an hPY-NLS motif interact with a negatively charged binding interface on the nuclear import factor Karyopherin ß 2 (Kap ß 2) [19]. Therefore, we reasoned that adding an acidic myc-tag immediately adjacent to the hPY-NLS in Rbfox3 may weaken or even abolish its interaction with Kap ß 2, resulting in altered steady-state sub-cellular distribution of the proteins.

We repeated our experiment with constructs containing N-terminal flag-tags (Figure 4). In this instance, Fox3v1 was predominantly nuclear, while Fox3v2 appeared to be exclusively nuclear and Fox3v3 appeared to be predominantly cytoplasmic; Fox3v2 and Fox3v3 differ *only* by the presence or absence of the 14 amino-acid C-terminal extension respectively, yet show starkly different steady-state sub-cellular distribution.

### All 3 Rbfox3 variants can regulate alternative splicing

**Figure 5:**
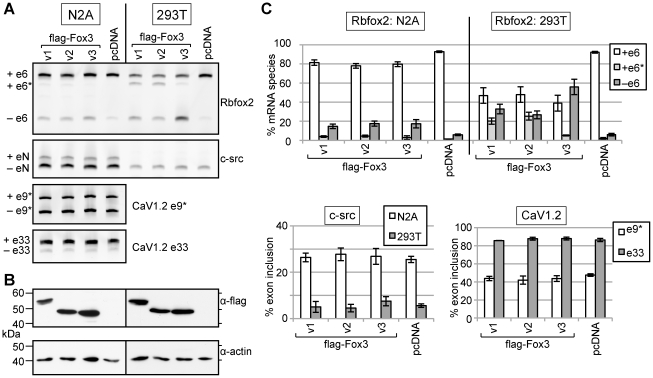
Rbfox3 variants all function to regulate alternative splicing, irrespective of steady-state sub-cellular localization. N2A or 293T cells were transiently transfected with Rbfox3 variants 1, 2 or 3 harboring N-terminal flag-tags, or pcDNA3 vector control. A. Alternative splicing of endogenous mRNAs encoding Rbfox2, c-src and CaV1.2 (alternative exons 9* and 33) was assayed by RT-PCR. Rbfox2 +e6* represents splicing from exon 5 to a cryptic exon downstream of exon 6. B. Western with anti-flag was used to confirm Rbfox3 overexpression, and anti-actin was used as a loading control. C. Quantification of the results shown in A and 2 additional experiments (i.e. n = 3); results are displayed as average +/− standard deviation. All Rbfox3 variants promoted skipping of Rbfox2 exon 6, but no change was seen in the splicing of c-src exon N or CaV1.2 exons 9* or 33 upon Rbfox3 overexpression.

The Fox family of RNA binding proteins are known to regulate alternative splicing of a number of pre-mRNAs [6], [8], [10], [12], [21]. Since pre-mRNA splicing occurs in the nucleus, we asked whether both nuclear and cytoplasmic forms of Rbfox3 could regulate alternative splicing. N2A and 293T cells were transiently transfected with N-terminal flag-tagged Rbfox3 constructs and harvested 24 hours post-transfection. The splicing of endogenous mRNAs was then assayed by RT-PCR using a FAM-labeled 3′ primer. Rbfox3v1 protein has previously been shown to inhibit the usage of exon 6 in Rbfox2 pre-mRNA (Rbfox3v2 and v3 have not been tested) [13]. Skipping of this exon creates an in-frame deletion in the RRM domain and results in production of a dominant negative isoform of Rbfox2 [13]. Consistent with this known role of Rbfox3v1, our data in Figure 5A shows that the addition of exogenous Rbfox3 results in skipping of exon 6 of Rbfox2. This effect is more pronounced in 293T cells, probably due to the higher transfection efficiency achieved in these cells (∼82% for 293T versus ∼50% for N2A cells). Surprisingly, the amount of exon 6 skipping is similar regardless of whether cells are transfected with the predominantly cytoplasmic Fox3v3 or the nuclear Rbfox3 variants v1 and v2; exon 6-containing mRNA was reduced from 92% of total Rbfox2 message for the empty vector control in 293T cells, to 47%, 48% and 39% for Fox3v1, v2 and v3 respectively (Figure 5A,C). This observation was confirmed by titration of the amount of plasmid transfected and subsequent quantification of Rbfox3 protein expression by fluorescent western. At similar expression levels of each of the Rbfox3 variants, the degree of Rbfox2 splicing regulation/alteration from baseline is comparable, as measured by the decrease in the steady-state amount of the productive +e6 pre-mRNA isoform (Figure S1). This robust splicing regulatory activity implies that either cytoplasmic Fox3v3 can influence the splicing of Rbfox2 pre-mRNA indirectly or, more likely, that Fox3v3 can shuttle between the nucleus and cytoplasm and regulate splicing directly.

Although all three Rbfox3 variants tested robustly repressed inclusion of Rbfox2 exon 6 in 293T cells, the nature of the alternative products detected is different (Figures 5 and S1). Fox3v1 and v2 transfected 293T cells harbored significant amounts of a third mRNA species that includes a cryptic exon derived from intron 6 of the Rbfox2 pre-mRNA that we have called exon 6*. Exon 6* is 74 nt in human 293T cells, and 71 nt in mouse N2A cells, but in both cases introduces a premature stop codon which would result production of a truncated protein or, more likely, target the mRNA for NMD (see below). This exon 6*-containing mRNA accounts for 20% and 25% of total Rbfox2 mRNA when Fox3v1 and v2 are transfected, respectively, compared to only 5% for Fox3v3 and 2% for pcDNA3. In contrast, in cells transfected with the cytoplasmically localized Fox3v3, 56% of Rbfox2 mRNA excludes both exon 6 and exon 6*, compared with 33% for Fox3v1, 27% for Fox3v2 and 6% for pcDNA3. Rbfox3 transfected N2A cells show a similar trend, although the magnitude of the changes is lower.

Interestingly, expression of the predominantly cytoplasmic C-terminal myc-tagged Fox3 variants in 293T cells also resulted in changes to Rbfox2 pre-mRNA splicing which most closely resembled those induced by Fox3v3 (Figure S1). By again comparing samples with similar Rbfox3 protein expression levels, we observed that the cytoplasmic, myc-tagged Fox3v1 and v2 proteins were as effective in causing skipping of Rbfox2 exon 6 (reduction to approximately 50% of total message) as their flag-tagged nuclear counterparts. However, unlike the flag-tagged variants, the amount of +e6* containing-mRNA detected accounted for less than 10% of the total Rbfox2 message. Again, we are unable to conclude whether these myc-tagged variants are affecting Rbfox2 splicing indirectly in the cytoplasm, or instead retain the ability to be imported to the nucleus.

We also analyzed splicing of additional reported targets of Fox protein-dependent splicing regulation, namely c-src and CaV1.2 [6], [22]. Upon overexpression in N2A cells, Rbfox1 and Rbfox2 have been shown to enhance the inclusion of a neuron-specific exon N1 in endogenous c-src message, and minigene assays have revealed that this splicing activation is dependent on Fox protein binding to UGCAUG elements downstream of exon N1 [6]. Rbfox3 has not been assayed as regulator of c-src alternative splicing. Interestingly, we did not detect a change in N1 exon inclusion with any of the Rbfox3 protein variants when overexpressed in N2A cells (293T cells do not exhibit any measurable inclusion of the N1 exon with or without the Rbfox3 proteins). Thus, either c-src N1 splicing cannot be regulated by Rbfox3, or the levels of Fox proteins required to regulated c-src splicing are much higher than those required to regulate Rbfox2 exon 6 splicing, and were insufficient in our experiments.

Overexpression of Rbfox1 and Rbfox2 has been shown to repress exon 9* but enhance exon 33 splicing in the CaV1.2 voltage-gated calcium channel pre-mRNA [22], and Rbfox3 has been shown to enhance exon 33 splicing (but only in a minigene context) [13]. Again, we did not see any alterations in splicing of CaV1.2 exons 9* or 33 in our Rbfox3 overexpression experiments. Thus we can conclude that the c-src and CaV1.2 pre-mRNAs are either weaker targets of Rbfox3-dependent splicing regulation than the Rbfox2 pre-mRNA in these cell lines, or not targets at all.

### Rbfox3 promotes nonsense-mediated decay of Rbfox2 transcripts

**Figure 6:**
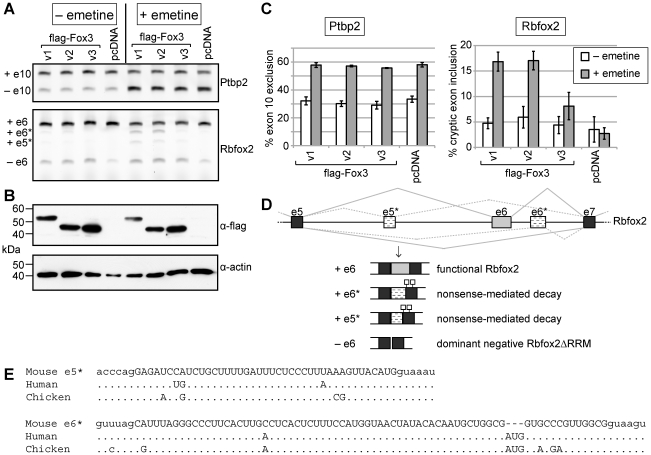
Rbfox3 variants enhance inclusion of cryptic exons in Rbfox2, leading to nonsense-mediated decay. N2A cells were transiently transfected with Rbfox3 variants 1, 2 or 3 harboring N-terminal flag-tags, or pcDNA3 vector control, and treated with emetine to inhibit NMD. A. Alternative splicing of endogenous mRNAs encoding Ptbp2 and Rbfox2 was assayed by RT-PCR. Enhanced exclusion of Ptbp2 exon 10 was used as a positive control for emetine treatment. Rbfox2 spliced products are described in D. B. Western with anti-flag was used to confirm Rbfox3 overexpression; anti-actin was used as a loading control. C. Quantification of the results shown in A and 2 additional experiments (i.e. n = 3); results are displayed as average +/− standard deviation. D. Schematic representation of alternative splicing of the Rbfox2 message around exon 6, indicating the relative positions of cryptic exons 5* and 6*. The functional outcomes for the resulting spliced mRNA products are depicted below; inclusion of exon 5* or exon 6* results in the presence of 2 in-frame nonsense codons in exon 7, and destruction of these messages by nonsense-mediated decay. E. The sequences of mouse Rbfox2 exons 5* and 6* (upper case) and surrounding intronic sequence (lower case) are shown aligned to the corresponding regions from human and chicken.

Since NMD requires active translation, treatment of mammalian cells with pharmacological inhibitors of translation results in the accumulation of transcripts normally targeted for NMD [23]. To further investigate whether Rbfox2 mRNAs including exon 6* are subject to NMD we treated N2A cells transiently transfected with our flag-tagged Rbfox3 constructs with the translation inhibitor emetine. We assayed skipping of exon 10 of polypyrimidine tract binding protein 2 (Ptbp2) by RT-PCR as a positive control for emetine treatment (Figure 6) since the isoform lacking exon 10 harbors a premature termination codon, and has been shown to accumulate when the NMD pathway is blocked [24], [25]. Emetine treatment alone had no effect on the relative levels of the different exon 6 Rbfox2 mRNA isoforms present in cells transfected with the empty pcDNA vector. However, N2A cells transfected with exogenous Rbfox3 showed a marked increase in accumulation of Rbfox2 mRNA containing exon 6* after treatment with emetine. Furthermore, this treatment revealed yet another mRNA species, this time containing a cryptic exon derived from Rbfox2 intron 5 that we have called exon 5* (Figure 6). Emetine treatment increases the abundance of transcripts containing either of these cryptic exons from 5% to 17% in cells expressing Fox3v1, from 6% to 17% for Fox3v2, and from 4% to 8% for Fox3v3. Inclusion of either exon 6* or exon 5* (which is 41 nt) alters the reading frame of the mRNA introducing two premature termination codons in the downstream exon, exon 7, as depicted in Figure 6D. Thus our data shows that Rbfox3 regulates alternative splicing of Rbfox2 to enhance the production of mRNA species that are targeted for NMD. This is likely to be a phylogenetically conserved mechanism of Fox family autoregulation since both of these cryptic exons and their splice sites are highly conserved amongst mammals, and in birds as shown in Figure 6E.

### Rbfox3 sub-cellular localization is not CRM1/exportin 1-dependent

**Figure 7:**
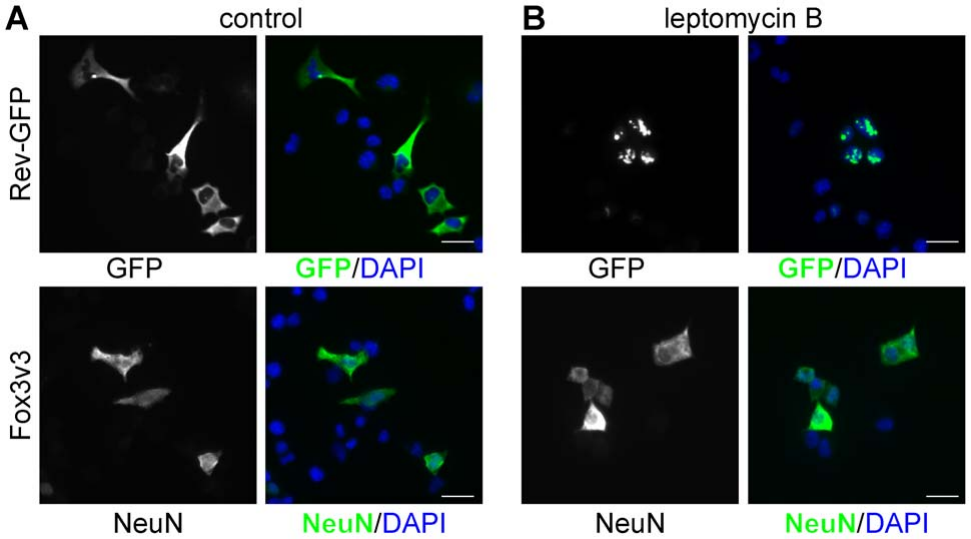
NeuN (Rbfox3) nuclear export is not Crm1/exportin 1-dependent. Rev-GFP fusion protein or Rbfox3 variant 3 with an N-terminal flag-tag were expressed in N2A cells and (A) left untreated or (B) treated with 10 ng/ml leptomycin B for 4 hrs prior to fixation and staining with anti-NeuN and DAPI, or just DAPI in the case of Rev-GFP. Leptomycin B treatment caused Rev-GFP to be retained in the nucleus, but caused no change in the sub-cellular localization of Fox3v3.

As shown above, Rbfox3v3 exhibits predominantly cytoplasmic steady-state sub-cellular localization, but is capable of regulating Rbfox2 alternative splicing to a similar degree as the nuclear-localized variants Rbfox3v1 and v2. In a preliminary attempt to determine if Rbfox3v3 actively shuttles between the nucleus and cytoplasm, we treated N2A cells transiently transfected with Rbfox3v3 with leptomycin B (LMB) to inhibit CRM1/exportin 1-dependent nuclear export [26]–[28]. Figure 7 shows that while the positive control Rev-GFP was retained in the nucleus after LMB treatment, no change was seen in the cytoplasmic localization of Fox3v3. This implies that either Fox3v3, which lacks a complete hPY-NLS, (1) is not capable of entering the cell nucleus or (2) can enter the nucleus, but is exported via a CRM1-independent mechanism. Interestingly, although immunofluorescence microscopy shows that the steady-state localization of Fox3v3 is predominantly cytoplasmic, a portion of the protein appears to be present in the nucleus of HeLa and N2A cells (see Figures 4 & 7); this observation, along with the ability of Fox-3v3 to regulate Rbfox2 exon 6 skipping, suggests that Fox-3v3 likely does shuttle, but confirmation of this hypothesis will need further study.

## Discussion

The target of the neuronal marker antibody anti-NeuN has recently been identified as the RNA binding protein Rbfox3 [5]. We have confirmed and extended this finding by IP and MS of *both* bands of the NeuN 45–50 kDa doublet. Like NeuN, Rbfox3 mRNA is expressed exclusively in post-mitotic neurons in mouse brain [5], [11], and we have shown that Rbfox3 mRNA is barely detectable in undifferentiated P19 cells, and strongly upregulated after retinoic acid-induced neuronal differentiation. Furthermore, we have demonstrated that Rbfox3 protein isoforms expressed from transfected cDNA vectors correspond in apparent molecular weight to the major protein species of NeuN from mouse brain.

Specifically, alternative splicing of exon 12 produces Rbfox3 proteins that differ by approximately 5 kDa, accounting for the observed anti-NeuN doublet by western; a second alternative splicing event at the C-terminus of Rbfox3 produces protein isoforms that differ by 1.4 kDa, likely accounting for the ‘doublet of doublets’ that can often be resolved with a SDS-PAGE gel (and subsequent western) of sufficient resolution [1], [2]. Proteins with and without this C-terminal extension have calculated pI values of ∼8.1 and 6.8 respectively, so this splicing event also likely accounts for the separation of both the 45 and 50 kDa bands into two major anti-NeuN immunoreactive spots after isoelectric focusing and PAGE observed by Lind *et al.* [1].

Given Rbfox3 is indeed NeuN, why did we fail to detect it in our original lambda expression screen? Lind *et al.* [1] argued that NeuN is a phosphoprotein, and while not necessarily directly phosphorylated, the anti-NeuN epitope may be phospho-dependent, which could prevent recognition in a bacterial system. However, we mapped the antibody binding site in Rbfox3 to a 20 amino acid region necessary and sufficient for anti-NeuN binding. The fact that we achieve recognition in the context of an autologous protein (GST) implies that the anti-NeuN epitope is linear, and is unlikely to require a particular structural context for anti-NeuN recognition. While we cannot rule out the possibility that the NeuN epitope is directly phosphorylated even when expressed as a fusion with GST, we believe the most likely explanation for the failure of the expression screen is that the epitope in Rbfox3 lies at the extreme N-terminus of the protein. The lambda screen requires that cloned cDNAs be in-frame with an N-terminal fragment of β-galactosidase; we speculate that the cDNA copies of Rbfox3 were either too short to contain the NeuN epitope, or too long, and thus could contain Rbfox3 5′ UTR sequence, which has a number of in-frame stop codons. However, our screen did identify a potential anti-NeuN cross-reacting protein, R3hdm2. We suggest that R3hdm2 may also be a potential contributor to the anti-NeuN signal in some instances given that overexpressed R3hdm2 can be detected by anti-NeuN in immunofluoresencent microscopy (not shown) and by western, and we observed a series of anti-NeuN immunoreactive bands of the appropriate size to be R3hdm2 that were specifically immunoprecipitated with anti-NeuN.

### Fox family cross-regulation

In this study, we found that Rbfox3, especially the nuclear splice variants v1 and v2, in addition to promoting skipping of exon 6 of Rbfox2, can enhance inclusion of two cryptic exons, which we have called exon 5* and exon 6*. In both instances, this introduces a premature stop codon in the Rbfox2 mRNA. Inhibition of the NMD pathway in mammalian cells resulted in further accumulation of these mRNA species indicating that they are targets of NMD. A close look at previously published data reveals that Rbfox1 may also have a similar activity on Rbfox2 [13]. The outcome of this cryptic splicing *in vivo* would be to downregulate Rbfox2 expression in Rbfox3 (and Rbfox1) expressing cells.

Alternative exons that induce NMD have been found to be particularly frequent in genes encoding splicing regulators [24]. A number of RNA binding proteins, such as Ptbp1 and SC35 directly influence their own pre-mRNA splicing to introduce premature termination codons and induce NMD of these mRNA isoforms, contributing to the maintenance of homeostasis of the levels of splicing regulators within cells [14], [15]. In addition, paralogous members of splicing regulator families have been shown to cross-regulate the levels of other family members by a similar mechanism. For example, hnRNP L enhances the inclusion of a short cassette exon containing a premature termination codon in its own message, as well as enhancing the inclusion of an analogous “poison” exon in closely related hnRNP L-like [29]. Similarly, Ptbp1 promotes exon skipping in its own, and closely related Ptbp2 messages, again leading to NMD of the resulting mRNA isoforms [30], [31]. In both of these instances, this cross-regulation contributes to reciprocal expression of these proteins in varying cell types.

Fox proteins have been previously shown to auto- and cross-regulate their production and function by enhancing skipping of a conserved cassette exon resulting in the expression of dominant negative ΔRRM proteins [13]. Here we provide the first evidence that alternative splicing-associated NMD also regulates Fox family cross-regulation. The existence of two conserved, complementary mechanisms for achieving cross- (and auto-) regulation of this family of RNA binding proteins imply that the absolute levels and balance of family members must be vitally important to cell function. Furthermore, our elucidation of the mechanism of regulation of Rbfox3 sub-cellular distribution suggests that different neuronal subtypes, which display varying ratios of nuclear/cytoplasmic anti-NeuN signal, express different Rbfox3 protein variants. In contrast to what has been reported for Rbfox1 [18], [32], we found that the cytoplasmic Rbfox3 isoform (v3) did not have diminished splicing regulatory activity. In fact, we observed functional differences in the splicing regulation by individual Fox proteins. Consequently, we predict that different neuronal subtypes will exhibit differences in the alternative splicing of target pre-mRNAs regulated by Rbfox3 and its family members, depending on their exact complement of Fox protein isoforms.

The identification of NeuN as Rbfox3 provides one more fascinating example of a RNA binding protein that is exquisitely neuron-specific, such as the Nova proteins, and most members of the Hu family, or neuronally-enriched, such as Ptbp2 (nPTB), and members of the CELF-Bruno family [33]–[38]. The recent development of CLIP, a technique that can specifically identify *in vivo* RNA target sites for any particular RNA binding protein, especially when combined with high-throughput sequencing methods, can allow one to rapidly move from the identification of a RNA binding protein to testing specific functional hypotheses regarding the protein's biological role in a neuron [39]–[42]. Indeed, RNA targets of Rbfox2 in embryonic stem cells have already been identified with such techniques [10]. One interesting but unanswered question is how do the Rbfox1, Rbfox2 and Rbfox3 splicing target sets compare? The CLIP technique is fundamentally dependent on having an antibody able to specifically IP the protein of interest in complex with bound RNAs. To that end, we have shown that anti-NeuN robustly and specifically IPs Rbfox3 (and not Rbfox1 or Rbfox2), and that the epitope is distant from the RRM, so antibody binding is unlikely to be occluded by crosslinked RNA. Therefore, a CLIP analysis of Rbfox3/NeuN should be highly successful.

## Materials and Methods

### Ethics statement

This study was carried out in strict accordance with the Australian code of practice for the care and use of animals for scientific purposes. The protocol was approved by the University of Adelaide Animal Ethics Committee (Permit Number: S-033-2008). Mice were killed by decapitation or carbon dioxide asphyxiation, and all efforts were made to minimize suffering.

### Library screen

We generated a cDNA expression library in Lambda Zap Express (Stratagene) from poly(A)^+^ RNA isolated from P0 mouse spinal cords with an estimated complexity of 1×10^6^ independent clones. The library was screened by probing plaque lifts on nitrocellulose filters with anti-NeuN mAb at a dilution of 1∶500. Secondary and tertiary screens were used to isolate individual reactive phage; phagemid rescues were performed according to protocols from Stratagene, and plasmid DNA containing the reactive cDNAs were sequenced.

### Plasmids

#### R3hdm2 constructs

The R3hdm2 coding sequence was amplified by PCR from the largest positive library clone, which encodes a protein identical to UniProt accession Q80TM6 isoform 3 (Q80TM6-3), and inserted into pET-41a^+^ (Novagen) via *Spe*I and *Hind*III to create plasmid FL. ΔABS is identical to FL except that it lacks amino acids 543–589 of Q80TM6-3; the deleted region was replaced with the amino acid sequence “GT” through creation of an internal *Kpn*I site by PCR. The ABS construct encodes amino acids 542–587 of Q80TM6-3 fused to the C-terminus of GST in pET-41a^+^. R46-myc was made by PCR amplification of the GST-ABS fusion from plasmid ABS and ligation into pCI (Promega) along with linkers to provide a 3′ myc-tag.

#### Rbfox3 constructs

Rbfox3 coding region was amplified from mouse brain cDNA by RT-PCR and cloned upstream of a myc-tag in pCI (Promega) or downstream of a flag-tag in pcDNA3 (Invitrogen). Variant 1 (v1) corresponds to NP_001034256, v2 to NP_001034257, and v3 to NP_001020102. Fox3Δ-myc is identical to Fox3v3-myc except that it lacks amino acids 5–20 of Fox3v3; the deleted region was replaced with the amino acid sequence “GSAP” by PCR. F20-myc encodes amino acids 2–21 of Fox3v3 fused to the C-terminus of GST in pCI; it also contains a C-terminal myc-tag.

#### Rev-GFP

HIV-1 Rev positive control for LMB treatment was plasmid pRev(1.4)-GFP-Rev from [43]. All constructs were confirmed by sequencing.

### Bacterial protein expression

Plasmids were transformed into BL21-CodonPlus (DE3)-RIPL competent cells (Stratagene), followed by standard IPTG (isopropyl-β-D-thiogalactopyranoside) induction. Bacterial cell pellets were lysed by boiling in SDS-load buffer prior to analysis by SDS-PAGE and western.

### Immunoprecipitation and mass spectrometry

Mouse brain nuclear extracts were prepared essentially as described previously [2]. Briefly, fresh brain tissue from C57BL6 mice was minced with a scalpel blade, then homogenized in 0.25% Triton X-100 in sucrose buffer (0.32 M sucrose, 1 mM MgCl_2_, 1 mM K-phosphate buffer pH 6.5) with a glass dounce homogenizer. Nuclei were pelleted by centrifugation (1,200× *g*, 10 min) and washed twice with sucrose buffer; this was considered the crude nuclear fraction. In some experiments, nuclei were further purified by resuspending the crude fraction in 2 M sucrose buffer and centrifuging through a 2.4 M sucrose cushion (53,000× *g*, 1 hour).

Nuclei were lysed in whole-cell extract buffer (WCE; 20 mM HEPES pH 7.5, 420 mM NaCl, 0.5% Igepal, 25% glycerol, 1.5 mM MgCl_2_) and incubated on ice for 30 min. The lysate was then diluted 1 in 5 with 20 mM HEPES pH 7.5, supplemented with 1 mM CaCl_2_ and treated with 150 units/ml micrococcal nuclease (Worthington) for 30 min. at 22°C with gentle mixing. EGTA was added to 2 mM and the lysate clarified by centrifugation (16,000× *g*, 10 mins). EDTA-free complete protease inhibitors (Roche), 1 mM Na_3_VO_4_ and 5 mM NaF were maintained throughout.

Anti-NeuN antibody, or mouse IgG antibody control was conjugated to protein-A sepharose beads (GE Healthcare) with dimethyl pimelimidate (DMP) as described previously [1] except that antibody binding was performed in the presence of 1 mg/ml BSA, and the DMP crosslinking incubation was performed twice prior to quenching the reaction with Tris-HCl pH 7.5. Crosslinked beads were washed twice with WCE buffer then resuspended in 750 µl mouse brain nuclear extract and incubated at 4°C for 1 hour with continuous rotation. The beads were pelleted by brief centrifugation and the supernatant fraction collected for analysis. Beads were then washed in WCE buffer followed by a total of 4 washes alternating between high and low salt wash buffers (high; 1 M NaCl, 20 mM HEPES pH 7.5, 0.5% Igepal, low; 1×PBS, 0.5% Tween-20). Beads were rinsed briefly with Na-phosphate buffer pH 7 and eluted with 1×Novex LDS load buffer (Invitrogen) for 10 min at 70°C. Samples were run on NuPAGE Novex 4–12% bis-tris gels with MES buffer (Invitrogen) under non-reducing conditions and either transferred to PVDF for western or stained with SilverQuest staining kit (Invitrogen).

Silver-stained protein bands were excised from the gel and destained immediately (within 1 hour). Proteins were digested in-gel with chymotrypsin or trypsin and the extracted peptides were chromatographed and analyzed using an HCT Ultra 3D-Ion-Trap mass spectrometer (Bruker Daltonik GmbH). MS and MS/MS spectra were subjected to peak detection using DataAnalysis (version 3.4, Bruker Daltonik GmbH) then imported into BioTools (version 3.1, Bruker Daltonik GmbH). Here, the MS/MS spectra were submitted to the in-house Mascot database-searching engine (version 2.2, Matrix Science).

### Tissue culture and Immunofluorescence

HeLa, 293T and N2A cells were propagated in DMEM supplemented with 10% fetal calf serum (FCS). For immunoblots, cells were transfected in 6-well plates with 0.5–1.0 µg DNA using Fugene 6 (Roche) for 24 hours. Cells were then washed with 1×PBS and lysed in WCE buffer with complete protease inhibitors (Roche), 1 mM Na_3_VO_4_, 5 mM NaF and 0.2 mM EDTA. Lysates were clarified by centrifugation (16,000× *g*, 10 mins) and 15 or 20 µg total protein was boiled in SDS load buffer.

For immunofluorescence, cells were grown on glass coverslips and transfected for 24 hours prior to fixation or treatment with 10 ng/ml LMB (Enzo Life Sciences) for 4 hours. Cells were fixed with 4% paraformaldehyde in PBS for 10 min, permeabilized with 0.5% Triton X-100 in PBS for 10 min and blocked with 10% FCS in PBST (1×PBS, 0.1% Triton X-100) for 1 hour. Cells were incubated with anti-NeuN 1∶150 in block for 1 hour, washed 3 times with PBST, then incubated with FITC anti-mouse secondary antibody (Jackson Immunolabs) 1∶200 in block. Cells were washed 3 times with PBST, with 0.5 µg/ml DAPI included in the second wash and mounted with ProLong Gold (Invitrogen). Images were acquired on a Zeiss Axioplan 2 using AxioVision software.

For target splicing assays, 293T cells and N2A cells were transfected in 12-well plates with 1.0 µg DNA using Fugene HD (Roche) for 24 hours prior to harvesting as discussed below. Emetine treatment was performed by adding 100 µg/ml emetine to the medium 18 hours after cell transfection, and 10 hours prior to harvesting.

P19 cells (ATCC) were propagated in α-MEM supplemented with 10% FCS. For differentiation, 1×10^6^ cells were transferred to 100 mm bacterial petri dishes in α-MEM supplemented with 5% FCS and 0.5 µM all-trans retinoic acid (RA, Sigma) for 4 days, with a medium change at day 2. At day 4, aggregates were treated with trypsin and DNaseI and plated onto poly-D-lysine coated tissue culture dishes in α-MEM, 10% FCS without RA and cultured for a further 3 days.

### Western blots

Samples were separated on 12% tris-glycine gels, transferred to PVDF and immunoblotted using mouse monoclonal anti-GST-tag 1∶2000 (Novagen 71097), anti-myc-tag (9B11, Cell Signaling) at 1∶1000, anti-HuR (3A2, Santa Cruz) at 1∶500, anti-NeuN (MAB377, Chemicon) at 1∶2000 for bacterial samples or 1∶500 for eukaryotic samples, anti-flag-tag (Sigma F7425) 1∶1000, anti-actin (Sigma A2066) 1∶1000. Detection was performed using horseradish-peroxidase-conjugated secondary antibodies (Jackson Immunolabs) and ECL using Western-Lightning-Plus (Perkin Elmer).

### RNA extractions, northern hybridization and RT-PCR

Total RNA was extracted from mouse tissues using a modified guanidine-acid phenol protocol [44]. Poly(A)^+^ RNA was subsequently purified using Oligo (dT)_25_ Dynabeads (Invitrogen) according to the manufacturer's instructions. 1–2 µg poly(A)^+^ RNA was resolved in a 1% agarose, 2.2 M formaldehyde gel, transferred to Hybond-N^+^ (Amersham), and fixed by UV-crosslinking. Northern hybridization was performed in Ultrahyb (Ambion) at 68°C. Antisense RNA probes were transcribed using Ambion's Strip-EZ RNA T7 kit, and labeled by incorporation of [α-^32^P]-UTP. The R3hdm2 probe template was amplified by PCR using primers F:gaacgaattcCGAGTGGCTAAAAAGAACTACGACC and R:agaaccaagcttAAATGGGAAAAGGGGAGGGCAG, cloned into pcDNA3 via the *Hind*III and *Eco*RI sites, and linearized with *Eco*RI. The β-actin probe was transcribed from plasmid pTRI-β-Actin-Mouse (Ambion). The blot was visualized by Typhoon PhosphorImager and quantified using ImageQuant.

Total RNA was purified from tissue culture cells using the RNeasy Mini kit system (Qiagen). Total RNA was reverse transcribed using random hexamers and SuperScript III (Invitrogen). Rbfox3 and gapdh products were amplified (28 cycles) using Taq polymerase (Invitrogen) and visualized in agarose gels with ethidium bromide. The primers were as follows: Rbfox3 e7-F, TTTAACGAGCGGGGCTCCAAG; e15A-R, TTCATGGTCCGAGAAGGAGACG; e15-R, GGTCTCTTGCTAGTAGGGGGTGAAG; e14-R, CATGGTTCCGATGCTGTAGG; Gapdh F, CGTCCCGTAGACAAAATGGT; R, CACATTGGGGGTAGGAACACG.

Endogenous splicing targets were amplified for 22 cycles (Rbfox2, Ptbp2), or 25 cycles (c-src and CaV1.2) using Taq polymerase (Invitrogen) and FAM-labeled 3′ or 5′ primers. Products were separated on 6% native polyacrylamide-TBE gels, visualized using a Typhoon Trio scanner and quantified using NIH ImageJ. Primers for Rbfox2, mouse c-src and CaV1.2 were as published [13], [45], [22]. For human c-src the 5′ primer used was CTGTCCTTCAAGAAAGGCGAGC (1 nt change from the mouse primer). Primers for Ptbp2 were F, CTGGTGGCAATACAGTCCTGTTG; R, TGGTTCCCATCAGCCATCTG. All PCR products were confirmed by gel extraction and sequencing.

## Supporting Information

Figure S1 **All myc-tagged and flag-tagged Rbfox3 variants function to regulate alternative splicing, irrespective of steady-state sub-cellular localization.** 293T cells were transiently transfected with increasing amounts of Rbfox3 variants 1, 2 or 3 harboring N-terminal flag-tags, or C-terminal myc-tags in 12-well plates. Empty vector was also added such that each well received 2 µg of plasmid. A. Upper panel: alternative splicing of endogenous Rbfox2 mRNA was assayed by RT-PCR. Lower panel: equal amounts of protein extract were separated by SDS-PAGE and immunoblotted with anti-NeuN and FITC-conjugated secondary antibody. The blots were visualized using a Typhoon Trio scanner and quantified using NIH ImageJ. The two gels were run back-to-back and processed together to enable quantification of the relative NeuN amounts; lane 1 was set to 1 and subsequent lanes are displayed as the fold-change from this value. Numbers in bold italics correspond to the samples used for quantification in B. B.Quantification of the RT-PCR results shown in A in bold. Only samples with similar levels of NeuN protein (2 to 3-fold above the amount in the first lane) are graphed, along with the controls. All Rbfox3 variants, whether flag- or myc-tagged, promoted skipping of Rbfox2 exon 6 to a similar degree. However, the inclusion of cryptic exon e6* was markedly higher after transfection of Rbfox3 protein variants which display nuclear steady-state subcellular distribution (namely flag-Fox3v1 and v2).
(TIF)
