# Supplementary figures and images for: NeuN/Rbfox3 Nuclear and Cytoplasmic Isoforms Differentially Regulate Alternative Splicing and Nonsense-Mediated Decay of Rbfox2
Source: PLoS One. 2011 Jun 29;6(6):e21585. doi: 10.1371/journal.pone.0021585 (PMC3126832; doi:10.1371/journal.pone.0021585)

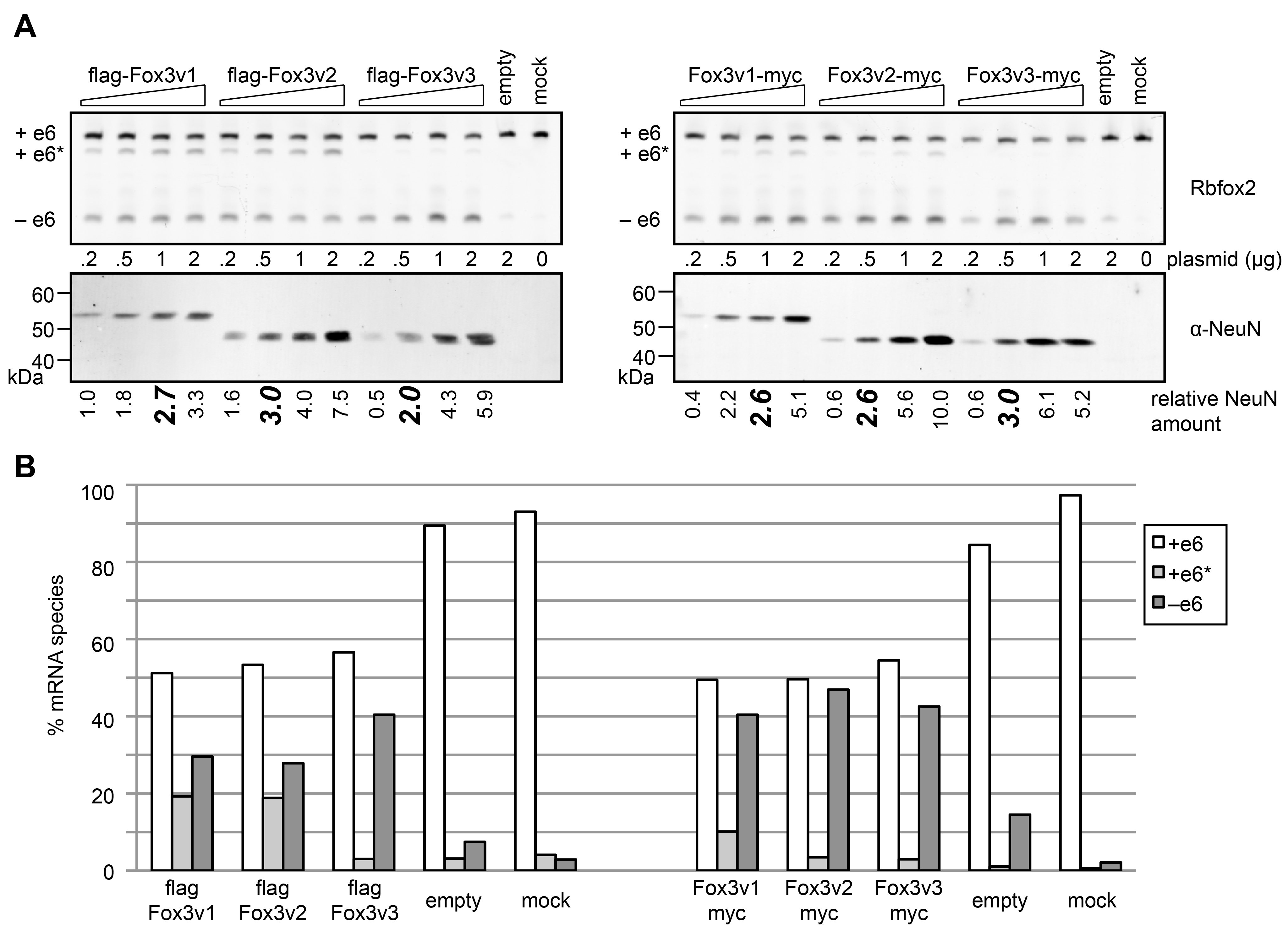

Supplement: Figure S1 — All myc-tagged and flag-tagged Rbfox3 variants function to regulate alternative splicing, irrespective of steady-state sub-cellular localization. 293T cells were transiently transfected with increasing amounts of Rbfox3 variants 1, 2 or 3 harboring N-terminal flag-tags, or C-terminal myc-tags in 12-well plates. Empty vector was also added such that each well received 2 µg of plasmid. A. Upper panel: alternative splicing of endogenous Rbfox2 mRNA was assayed by RT-PCR. Lower panel: equal amounts of protein extract were separated by SDS-PAGE and immunoblotted with anti-NeuN and FITC-conjugated secondary antibody. The blots were visualized using a Typhoon Trio scanner and quantified using NIH ImageJ. The two gels were run back-to-back and processed together to enable quantification of the relative NeuN amounts; lane 1 was set to 1 and subsequent lanes are displayed as the fold-change from this value. Numbers in bold italics correspond to the samples used for quantification in B. B.Quantification of the RT-PCR results shown in A in bold. Only samples with similar levels of NeuN protein (2 to 3-fold above the amount in the first lane) are graphed, along with the controls. All Rbfox3 variants, whether flag- or myc-tagged, promoted skipping of Rbfox2 exon 6 to a similar degree. However, the inclusion of cryptic exon e6* was markedly higher after transfection of Rbfox3 protein variants which display nuclear steady-state subcellular distribution (namely flag-Fox3v1 and v2). (TIF) [file pone.0021585.s001.tif]
